# Retraction: Potential malignant transformation in the gastric mucosa of immunodeficient mice with persistent *Mycoplasma penetrans* infection

**DOI:** 10.1371/journal.pone.0241463

**Published:** 2020-10-22

**Authors:** The PLOS ONE Editors

Following publication of this article [1], concerns were raised regarding the histopathology and western blot image data.

Specifically:

- There are concerns about the labelling and interpretation of the image panels for hematoxylin-eosin (HE) staining of *Mycoplasma penetrans (*Mpe) infected mice in Fig 2C and Fig 2D. Evaluation of these images by an independent Editorial Board member did not support the reported findings of precancerous gastric mucosal lesions. Review of replacement images provided did not resolve the concerns about the accuracy of the experimental group labelling or interpretation of the image data.
- The underlying blot images provided as Supporting Information on the original article do not show the western blots used to compile the original published Figs 3–5, with the exception of the GAPDH panels for Figs 3 and 4.
- There are concerns about the appearance of the western blot panels:
  ○ In Fig 3, there are discontinuities in background appearance in the p21 and H-ras panels
  ○ In Fig 3, lanes 2–4 of the GAPDH panel appear similar to lanes 1–3 of the GAPDH panel in Fig 4; the labelling of the underlying GAPDH blots in the Supporting Information of the original article correspond to the GAPDH lane labels of Fig 3, but not Fig 4.
  ○ In Fig 5, lanes 3–4 of the Bcl-2 panel appear similar to lanes 3–4 of the GAPDH panel, and lanes 1–2 of the Bax panel appear similar to lanes 1–2 of the GAPDH panel.

The corresponding author has indicated that raw western blot images were modified in a manner inconsistent with the journal’s policies for blot and gel data, and apologizes for the breach of the journal’s policies on figure preparation.

In light of the concerns about the accuracy of the identification and interpretation of the histopathology data, and about the inappropriate modification of western blot image data, the *PLOS ONE* Editors retract this article.

SC, LL, Yuxue W agreed with the retraction. DS, Yadong W, LZ either could not be reached or did not reply directly. SC, LL, Yuxue W apologize for the issues with the published article.
